# Sleep and its relation to health-related quality of life in 3–10-year-old children

**DOI:** 10.1186/s12889-021-11038-7

**Published:** 2021-06-02

**Authors:** Anna Lena Sundell, Charlotte Angelhoff

**Affiliations:** 1 Department of Pediatric Dentistry, Institute for Postgraduate Dental Education, Jönköping, Sweden; 2 Centre of Oral Health, School of Health and Welfare, Jönköping University, Jönköping, Sweden; 3 Crown Princess Victoria’s Child and Youth Hospital, and Department of Biomedical and Clinical Sciences, Linköping University, Linköping, Sweden

**Keywords:** Child, Child preschool, Family, Primary health care, Sleep, Quality of life

## Abstract

**Background:**

Considering the reports of increasing sleep problems in children, affecting health and well-being in young children and their families, we found it important to gain more knowledge about sleep and its correlation to health-related quality of life (HRQoL) in young, healthy children. The aims with this study were to describe sleep quality, sleep duration, and HRQoL in healthy 3–10-year-old children and to test associations between children’s sleep and HRQoL.

**Methods:**

Parents of 160 children (average age: 6.9 years, SD ±2.2) participated in the study. Sleep onset problems (SOP), sleep maintenance problems (SMP), and sleep duration were measured by the Pediatric Insomnia Severity Index (PISI). KIDSCREEN-27 was used to measure HRQoL in five dimensions: physical well-being, psychological well-being, autonomy and parent relation, social support and peers, and school environment.

**Results:**

The average score was 2.2 for SOP (SD +/− 2.2) and 1.3 for SMP (SD +/− 1.6). Few children (2%) were reported to sleep less than 8 h per night. Younger children had statistically significant higher SOP and SMP than older children. Correlations were found between SOP and poor psychological well-being (*p* < 0.05, *ρ* = − 0.16), and between SMP and poor physical wellbeing (*p* < 0.05, *ρ* = − 0.16), psychological well-being (*p* < 0.05, *ρ* = − 0.21), poor school environment (*p* < 0.01, *ρ* = − 0.29), autonomy and parent relation (*p* < 0.05, *ρ* = − 0.16), and poor social support and peers (*p* < 0.05, *ρ* = − 0.19).

**Conclusion:**

Children’s sleep associates with health-related quality of life and needs to be acknowledged in child health care settings and schools.

## Background

Adequate sleep duration, good sleep quality, regularity, and the absence of sleep disruptions are important factors for healthy sleep in children [1, 2]. Adolescents have recently described good sleep in terms of sleep duration, bedtime routines, short sleep onset latency, absence of nocturnal awakenings, and waking up feeling rested and joyful [3], which is in line with the definition of sleep quality described by Ohayon et al. [4]. According to the Consensus Statement of the American Academy of Sleep Medicine, pre-school children (3–5 years old) need 10 to 13 h of sleep and school-aged children (6–10 years old) need 9 to 12 h of sleep on a regular basis to maintain optimal health [2].

No previous study of prevalence and trend of sleep problems in healthy, young children has been found, but studies have reported increasing sleep problems in children and adolescents [5–7], leading to physical, psychological, and cognitive problems [6, 8, 9]. Sleep problems influence emotions and regulation of neurobiological behavioural and cognitive processes, mainly through behavioural tendencies and neurological changes. Consequently, insufficient sleep impacts emotional reactivity, leading to more negative and less positive emotions [10, 11], but also to impaired executive functions, which negatively affects learning abilities and school performance [9, 12].

Further, sleep is associated with health-related quality of life (HRQoL). However, there is a lack in the literature of these associations in young children. A recently published study of 9–11-year-old children in 12 countries reveals that self-reported poor sleep in children is associated with low HRQoL, but it found no associations between device-based measured sleep and HRQoL [13]. Magee et al. [14] report in an Australian cohort study that disordered sleep and minor sleep disturbance in 10- and 11-year-old children relate to poorer HRQoL, which worsen over time. Price et al. [15] report weak and inconsistent correlations between sleep duration and HRQoL in 4–9-year-old children, suggesting that parameters other than sleep duration could be more meaningful to understand optimal sleep in children.

According to the World Health Organization [16], HRQoL is the personal judgment of one’s health and diseases, including a multidimensional assessment of a person’s satisfaction with life and the perceptions of his or her position in life in different contexts. Previous studies of children’s sleep or HRQoL have mostly focused on different health problems and diagnoses such as epilepsy [17], asthma [18], atopic dermatitis [19] and type 1 diabetes [20]. No study is found of sleep and HRQoL in young healthy children.

Considering the reports of increasing sleep problems in healthy children, affecting health and well-being in young children and their families, we found it important to gain more knowledge about sleep and its correlation to HRQoL in a healthy population of young children in Sweden. The aims of the current study were to describe sleep quality, sleep duration, and HRQoL in healthy 3–10-year-old children and to test associations between children’s sleep and HRQoL.

## Methods

### Design

This is an explorative and descriptive study with a cross-sectional design. The study was approved by the Regional Ethics Committee for Medical Research (Dnr 2018/175–31) in accordance with the Helsinki declaration [21].

### Participants and procedure

A consecutive sample of Swedish-speaking parents of children between 3 and 10 years old with no major health problems were asked to participate when visiting child health care centres and public dental services for the child’s regular health check-up. To achieve a representative population, we selected health care centres and public dental clinics with catchment areas from large cities as well as small towns at the countryside in two regions in South Eastern Sweden. It is not known how many parents the staff excluded due to language difficulties or health problems or how many parents declined to participate. After providing informed consent, the parents answered the Pediatric Insomnia Severity Index (PISI) and KIDSCREEN-27 proxy version for their children. In cases where the parent had more than one child between 3 and 10 years old, they were asked to fill out questionnaires for the siblings as well. If they accepted, they received another coded envelope with questionnaires for the sibling. The coded, completed forms were placed in a postage-paid envelope and returned to the authors. Data were collected between September 2018 and January 2019.

### Measurements

Sleep was estimated by the six-item Swedish version of the PISI, a parent-proxy questionnaire for children between 3 and 10 years old. The items follow the ICSD-II general criteria for insomnia, including difficulties falling asleep, difficulties maintaining sleep, and daytime impairment. Five of the items are scored on a six-point Likert-type scale (0 = never, 5 = always), and total hours of sleep on most nights are rated on a six-point scale (0 = 11–13 h, 5 = less than 5 h). Higher values indicate more sleep problems. The questionnaire refers to the children’s sleep over the last week and consists of two dimensions of sleep difficulties: sleep onset problems (SOP) and sleep maintenance problems (SMP). The PISI is validity and reliability tested with good results for brief screening of insomnia [22, 23]. The validity and reliability test of the Swedish version of PISI was calculated on this sample with good results and have been formerly presented in Angelhoff et al. [23].

To measure various aspects of HRQoL in healthy children, we sought a general HRQoL instrument available in the Swedish language, with a reasonable number of items with a salutogenetic perspective, developed in accordance with the International Society for Pharmacoeconomics and Outcomes Research (ISPOR) Patient Reported Outcomes (PRO) Good Research Practices Task Force Report [24, 25]. KIDSCREEN-27 was developed as a self-report measurement applicable for healthy and chronically ill children and adolescents aged from 8 to 18 years in an intercountry collaboration network including 13 European countries; created after literature reviews, consultations with experts, and discussions in focus groups with children, and thereafter adjusted and tested as a proxy-version [26–28]. According to the developers of KIDSCREEN-27, obtaining responses via self-reports may not be practicable in very young children, but HRQoL may be ascertained via proxy reports [26]. Therefore, the proxy-version of KIDSCREEN-27 was considered to be appropriate for this study of children 3–10 years old.

The 27 items are scored on a five-point Likert-type scale (1 = no agreement at all, 5 = total agreement). The generic questionnaire includes five dimensions of HRQoL: physical well-being (level of physical activity, energy, and fitness), psychological well-being (positive emotions and satisfaction with life), autonomy and parent relation (perceived level of autonomy, interaction between child and parent, and feeling loved and supported), social support and peers (interaction between child and peers), and school environment (perception of cognitive capacity, learning, concentration, and feelings about school). The questions refer to the children’s HRQoL over the last week. Higher values indicate better HRQoL, and T-scores of 50 and SD ± 10 are regarded as normal. The measurement has acceptable reliability (Pearson’s *r* 0.61–0.74) and validity, as well as internal consistency (Cronbach’s α 0.79–0.84), in analyses including several European languages [27, 28]. In this sample, Cronbach’s α was 0.94. Availability and permission to utilize the Swedish version of KIDSCREEN-27 was granted by the copyright holder [27].

### Statistical analysis

Rasch measurement analysis was used for all five KIDSCREEN-27-dimensions and thereafter transformed to T-values, according to the manual [26]. Descriptive statistics are presented as means (m), standard deviations (SD), medians, interquartile range (Q1, Q3), frequencies (*n*), and percentage (%). Nonparametric tests were used, as both the PISI and KIDSCREEN-27 include qualitative variables. The Mann–Whitney U-test was performed to calculate differences between genders. Spearman’s rho (ρ) was used for comparison of dimensions of HRQoL and dimensions of sleep problems, gender, and age. A two-samples t-test was conducted to compare KIDSCREEN-27 between our sample and the European reference population, consisting of > 8000 children 8–18 years old, used in Ravens-Sieberer et al. [27].

All reported *p*-values were two-sided, and a *p*-value of < 0.05 was considered statistically significant. Data were processed using IBM SPSS statistics version 25.

## Results

Parents of 188 children gave consent to participate in the study, of whom 28 (15%) were excluded due to incorrectly filled out questionnaires. The final sample consisted of 160 children at an average age of 6.9 years old (SD ±2.2, age range 3.0 to 10.7 years), of whom 44% were girls. Four parents filled out questionnaires for more than one child: one family with two children and three families with three children. A review of the data from the siblings showed that the questionnaires had been answered individually for each child and could thus be included in the analyses.

Mean values on sleep outcomes are presented in Table 1. Fourteen children (9%) had trouble falling asleep at bedtime, and 25 children (16%) took longer than 30 min to fall asleep. Girls were reported to have statistically significant more problems falling asleep (m 2.67, SD ±2.3, *p* = 0.01) than boys (m 1.9, SD ±2.0). Eight children (5%) woke more than once during the night, two children had trouble returning to sleep after waking, and one child appeared sleepy during the day. Few children (*n* = 4, 2%) were reported to sleep less than 8 h.

**Table 1:** Description of the children’s sleep and health-related quality of life, and differences between girls and boys

	All children *n* = 160 mean (SD) median (Q1, Q3)	Girls *n* = 70 mean (SD) median (Q1, Q3)	Boys *n* = 90 mean (SD) median (Q1, Q3)	*P*-value^a^
Age	6.9 (2.2)	7.0 (2.1)	6.9 (2.3)	.17
	7 (5, 9)	7.4 (5.2, 8.4)	7.0 (5.0, 8.9)	
PISI				
Sleep onset problems	2.2 (2.2)	2.7 (2.3)	1.9 (2.0)	.01*
	2 (0, 3)	2 (0, 2)	1 (0, 2)	
Sleep maintenance problems	1.3 (1.6)	1.3 (1.5)	1.3 (1.7)	.99
	1 (0, 2)	1 (0, 2)	1 (0, 2)	
Total hours of sleep^b^	1.0 (0.6)	1.1 (0.7)	0.9 (0.5)	.06
	1 (1, 1)	1 (1, 1)	1 (1, 1)	
KIDSCREEN				
Physical well-being	54.6 (8.5)	54.0 (8.9)	55.1 (8.3)	.77
	52.7 (49.5, 59.4)	52.7 (38.3, 71.2)	55.9 (34.5, 71.2)	
Psychological well-being	55.2 (9.5)	54.8 (9.5)	55.5 (9.6)	.59
	52.4 (46.7, 58.3)	54.0 (40.0, 76.4)	56.0 (38.2, 76.4)	
Autonomy and parent relation	56.3 (9.2)	56.7 (10.2)	55.9 (8.4)	.26
	56.0 (51.1, 62.9)	56.0 (37.0, 79.1)	56.0 (37.0, 79.1)	
Social support and peers	55.4 (8.0)	54.7 (7.9)	56.0 (8.0)	.95
	52.6 (49.1, 56.1)	52.6 (42.9, 70.3)	52.6 (33.3, 70.3)	
School environment	58.1 (9.1)	59.6 (8.2)	56.9 (9.6)	.08
	55.4 (51.4, 70.7)	59.3 (29.4, 70.7)	55.4 (60.2, 70.7)	

* significant at *p* < .05

^a^ Mann–Whitney U-test

^b^ Six-point Likert-scale from 0 = 11–13 h to 5 = less than 5 h

A statistically significant correlation was found between SOP and total hours of sleep (*p* = 0.03, *ρ* = 0.17) — i.e., the more problems falling asleep, the fewer hours of sleep. Moreover, the child’s age correlated significantly with SOP (*p* = 0.03, *ρ* = − 0.17) and SMP (*p* = 0.02, *ρ* = − 0.18), indicating that the lower the age, the more sleep problems. When dichotomizing the children’s ages into two groups — pre-school (3–5 years) and school-aged (6–10 years) children — there was a statistically significant correlation in SOP (*p* = 0.02, *ρ* = − 0.19), but not in SMP. There was also a statistically significant correlation (*p* = 0.03, *ρ* = 0.18) between the child’s age and total hours of sleep (where higher rating indicates fewer hours of sleep), suggesting that sleep duration decreases with the child’s age. This correlation remained significant when we compared the dichotomized groups of pre-school and school-aged children (*p* = 0.03, *ρ* = 0.17).

Scores for the five dimensions of KIDSCREEN-27 are presented in Table 1. Nearly all children (*n* = 155, 97%) reported good, very good, or excellent general health. Three children had poor or fairly poor general health (item no. 1). There was a significant difference in the scores for the children in our study (m 56, SD ±7) and the European reference population (m 50, SD ±10, *t*[179] = − 10.6, *p* < 0.01) [25]. Age and gender were not statistically significant associated to HRQoL.

SOP was significantly correlated with poor psychological well-being, and SMP was significantly correlated with poor physical wellbeing, poor psychological well-being, poor school environment, poor autonomy and parent relation, and poor social support and peers (Table 2).

**Table 2:** Correlation coefficients ^a^ between the PISI and KIDSCREEN-27 (*n* = 160)

		Sleep onset problems	Sleep maintenance problems	Physical well-being	Psychological well-being	Autonomy and parent relation	Social support and peers
Sleep maintenance problems	*ρ*	,242^**^					
	*p*-value	,002	–				
Physical well-being	*ρ*	-,059	-,159^*^				
	*p*-value	,468	,049	–			
Psychological well-being	*ρ*	-,163^*^	-,206^**^	,553^**^			
	*p*-value	,041	,009	,000	–		
Autonomy and parent relation	*ρ*	-,072	-,163^*^	,536^**^	,528^**^		
	*p*-value	,384	,048	,000	,000	–	
Social support and peers	*ρ*	-,112	-,186^*^	,375^**^	,362^**^	,385^**^	
	*p*-value	,166	,021	,000	,000	,000	–
School environment	*ρ*	-,124	-,286^**^	,478^**^	,538^**^	,505^**^	,448^**^
	*p*-value	,127	,000	,000	,000	,000	,000

^*^ Correlation is significant at the 0.05 level (2-tailed)

^**^ Correlation is significant at the 0.01 level (2-tailed)

^a^ Spearman’s rho (*ρ*)

## Discussion

The main results of this study were the correlations between sleep and the HRQoL-dimensions psychological well-being, school environment, and social support and peers. It is difficult to say what comes first, sleep disturbances or poor HRQoL. Certainly, they are associated with each other and likely connected with each other like a vicious circle. Sleep disturbances in children have previously been reported to be associated with emotional problems such as anxiety [29], leading to late bedtimes, poor sleep duration, and frequent night awakenings [30, 31]. In a systematic review, longer sleep duration has been found to be related with better emotional regulation and quality of life in 5–17-year-old children [32].

The overall sleep duration for the children in the present study was 9–11 h per night, which is in line with general sleep duration recommendations [2, 33], but contrasts with other studies describing less sleep duration in the latest decennium [7, 34]. Longer sleep duration was seen in younger children compared to older. Biological rhythm of sleep and waking is regulated through both circadian and homeostatic processes, but also active and complex neurophysiologic processes that change over the life course, especially in the first 5 years, leading to longer sleep duration [6]. Furthermore, it could be expected that older children have later bedtimes than younger children. According to Norell-Clarke and Hagquist [7], there has been a change in bedtime over time, with later bedtimes, less sleep, and sleep onset difficulties in 11-year-old children in Sweden. However, the children in our study were below the age of 11 years and cannot be fairly compared to the above-mentioned study. The widespread possible answer (3 h) in the PISI (item no. 6: total hours of sleep on most nights) should also be taken into consideration. Therefore, the PISI may be more appropriate for screening sleep problems such as SOP and SMP, than measuring subjective sleep duration, i.e. total hours of sleep.

In present study, problems maintaining sleep were related to the HRQoL-dimension poor school environment, which includes cognitive capacity and feelings about school, as well as the dimension social relation with peers and friends. These results are in line with those reported by Gustafsson et al. [35], who report associations between daytime sleepiness and HRQoL, including school-related sub-score measured by the Pediatric Quality of Life Inventory, in 10–15-year-old Finnish school children. Several other studies have revealed the association between sleep and impaired school performance, academic achievement, attention, and learning motivation [36, 37]. Furthermore, peer victimization, including bullying, has been shown previously to be related to sleep problems [38–40]. The relation between peer victimization and sleep problems is stronger in younger than older children [38]. Being bullied is associated with symptoms of severe mental health problems and can have serious consequences over time [41, 42]. Direct questions about victimization or bullying are not included in KIDSCREEN-27, but the quality of the interaction between the child and peers as well as their perceived support are explored. Low scores are interpreted as feeling excluded and not accepted by peers [27]. Knowing that sleep is important to manage school as well as being able to interact with peers and friends, teachers, and school nurses should inquire about both the quality and quantity of the child’s sleep on a routine basis to promote health and well-being in school children, and advice and support should be offered to the children and their parents in following good sleep hygiene principles.

In the present study, the children had higher HRQoL than the European reference population [27]. This result is in concordance with a previous study of HRQoL in 5–10-year-old children in Sweden using the proxy-version KIDSCREEN-52 [43]. Only one child out of 10 was reported to have trouble falling asleep at night, and one out of six took longer than 30 min to fall asleep. This indicates that overall, healthy children in Sweden have a good sleep quality and a good HRQoL. Moreover, we found that the child’s age was associated with sleep problems; the lower the age, the more problems. It has been previously explained that sleep problems, such as nightmares, sleep terrors, and sleep walking, are more common in pre-schoolers compared to school-aged children [44, 45]. It is important to acknowledge children’s sleep as well as psychological well-being when meeting the child at regular health visits. Advice about calming bed routines and healthy sleep habits in combination with good sleep hygiene should be provided early to parents to help prevent sleep problems and manage sleep problems when they happen [6].

This study is one of few studies exploring healthy children’s sleep and its correlation to HRQoL. It provides valuable insight into the importance of sleep for psychological well-being, school, and social relations. However, there are some limitations that need to be mentioned. Only Swedish-speaking parents were included in the study, which excludes a large part of the Swedish population and limits the transferability to other countries and cultures. The methodology is subjective and based on parent-report. Heightened problems in younger children may be more reflective of parental problems or unreasonable expectations. Reduced problems in older children may reflect lack of parental awareness. In addition, there is no data on family bed-sharing. According to Welles-Nystrom [46], Swedish children often co-sleep with both their parents until school age. Increased sleep proximity may make parents more aware of their child’s sleep behaviours. Another potential weakness is that four parents filled out questionnaires for siblings, which may have contributed to Type II-errors.

A validation of the PISI to an objective assessment tool, such as actigraphy or polysomnography, would be of highest interest. Moreover, PISI does not have a cut-off for acknowledging symptoms of severe sleep problems. Future research should focus on determining a cut-off score, as presented in the adult version of the Insomnia Severity Index [47].

## Conclusion

This study highlights the importance of acknowledging children’s sleep in child health care settings and schools. The children’s HRQoL was related to the child’s sleep. We also found that the young healthy children in our study had few sleep problems and a good HRQoL. As sleep problems in children are increasing, the result of this study aims to attract attention to the importance of awareness of children’s sleep.

## Data Availability

The datasets used and analysed during the current study are available from the corresponding author on reasonable request.

## Abbreviations

HRQoL: Health-related quality of life
PISI: The Pediatric Insomnia Severity Index
SMP: Sleep maintenance problems
SOP: Sleep onset problems
